# Evaluation of a Multicore-Optimized Implementation for Tomographic Reconstruction

**DOI:** 10.1371/journal.pone.0048261

**Published:** 2012-11-06

**Authors:** Jose-Ignacio Agulleiro, José Jesús Fernández

**Affiliations:** 1 Supercomputing and Algorithms group, University of Almería, Almería, Spain; 2 National Centre for Biotechnology, National Research Council (CNB-CSIC), Madrid, Spain; University of Heidelberg Medical School, Germany

## Abstract

Tomography allows elucidation of the three-dimensional structure of an object from a set of projection images. In life sciences, electron microscope tomography is providing invaluable information about the cell structure at a resolution of a few nanometres. Here, large images are required to combine wide fields of view with high resolution requirements. The computational complexity of the algorithms along with the large image size then turns tomographic reconstruction into a computationally demanding problem. Traditionally, high-performance computing techniques have been applied to cope with such demands on supercomputers, distributed systems and computer clusters. In the last few years, the trend has turned towards graphics processing units (GPUs). Here we present a detailed description and a thorough evaluation of an alternative approach that relies on exploitation of the power available in modern multicore computers. The combination of single-core code optimization, vector processing, multithreading and efficient disk I/O operations succeeds in providing fast tomographic reconstructions on standard computers. The approach turns out to be competitive with the fastest GPU-based solutions thus far.

## Introduction

Tomographic reconstruction derives the three-dimensional (3D) structure of an object from a set of projection images acquired by means of some imaging process. This technique is central in many scientific and technical disciplines [1]. Electron tomography (ET), where the imaging device is an electron microscope, allows elucidation of the 3D structure at nanometric scale [2]–[5]. ET is playing an essential role in life sciences. It has already made possible a number of major breakthroughs in the last decade, which have provided invaluable information about the cell structure [6]–[11]. Tomographic reconstruction algorithms are the core of the technique. They combine the information contained in the projection images and yield the 3D structure. The projection images taken from the biological sample usually present a size in the range of 1024
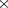
1024 to 2048
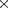
2048. The number of images commonly ranges from 60 to 200. The resulting 3D maps (also known as tomograms) may reach a size in the order of several GBytes (e.g. 2048
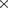
2048
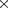
512 voxels). There exist several families of reconstruction algorithms that are commonly used in the field [2].

The computational complexity of the algorithms, along with the data size (number and size of the images), turns tomographic reconstruction into a computationally demanding problem. Historically, high performance computing (HPC) has been applied to cope with those demands in ET [12]. There have been proposals for supercomputers [13], distributed systems [14]–[16] and computer clusters [17]–[22]. Most of these implementations have approached linear speedup factors. Recently, the trend has turned towards exploitation of graphics processing units (GPUs) [23], and a number of approaches have been presented [24]–[29], including the use of multi-GPU or hybrid strategies [30]–[33], which have achieved outstanding speedup factors.

On the other hand, current stand-alone computers present tremendous power thanks to technological and architectural advances [34]. Specific features such as internal instruction-level parallelism, vector instructions, multiple computing cores as well as deeper and efficient memory hierarchies turn modern computers into extraordinary computing platforms, with impressive performance [35]. In the last few years, we have been optimizing tomographic reconstruction algorithms for these platforms. First we introduced the potential of vector processing and showed preliminary results [36]. Later we introduced our software package [37] (http://www.cnb.csic.es/%7ejjfernandez/tomo3d), which makes use of vector processing and multithreading to exploit the multiple cores available in modern computers. Our software is now being extensively used in the ET field [38]–[43]. However, a thorough explanation and assessment of the procedures behind is still lacking. Moreover, recently we have made additional, important improvements related to the disk I/O and dynamic load balacing, which contribute to further accelerate the reconstruction process.

In this work we present a detailed description and evaluation of our optimized implementation of tomographic reconstruction algorithms for modern multicore computers. This article is organized as follows. Section 2 reviews the most common tomographic reconstruction algorithms in the field of ET. Section 3 then describes in detail our approach to fast tomographic reconstruction. The experimental results are presented in Section 4, where different performance aspects (processing time; I/O; load balancing) are evaluated and comparisons with modern GPU approaches and a standard program are included. The last section provides some discussion and concluding remarks.

**Figure 1:**
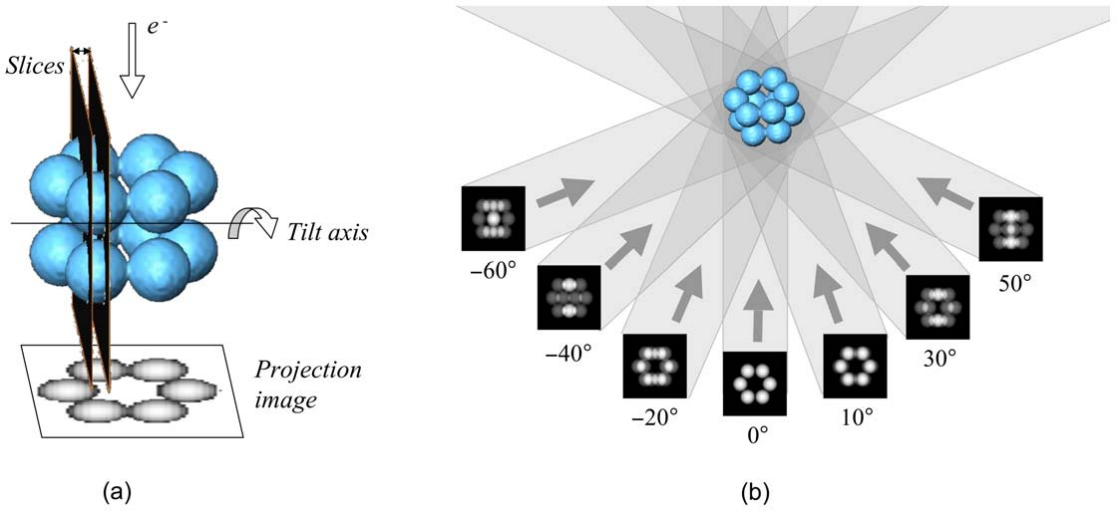
Image acquisition and tomographic reconstruction. (a) Single-tilt axis data acquisition geometry. The specimen is imaged in the microscope by tilting it over a typical range of [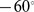,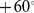] or [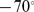, 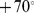] in small tilt increments. The specimen can be considered as being composed of slices perpendicular to the tilt axis, as sketched. Hence, every projection image holds information about all the slices. (b) Three-dimensional reconstruction from projections with backprojection. The projection images are projected back into the volume to be reconstructed.

### Overview of Tomographic Reconstruction

The standard data acquisition geometry in ET is the so-called single tilt axis [3], [4]. The specimen is placed within the electron microscope and a beam of electrons is shot towards it in a direction perpendicular to the single tilt axis. A projection image is formed and recorded by CCD cameras. The specimen is then tilted around the axis, and another beam of electrons is shot. This is sketched in Figure 1 (a). Typically, the process is repeated over a limited tilt range of [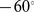, 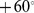], or [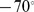, 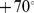], in small increments of 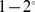. Electron dose must be kept within tolerable limits to prevent radiation damage to the specimen, which yields projection images with extremely low signal-to-noise (SNR) ratio. As a result of this data collection process, the so-called tilt series is obtained, which is made up of all the images acquired from the specimen at the different orientations.

**Figure 2:**
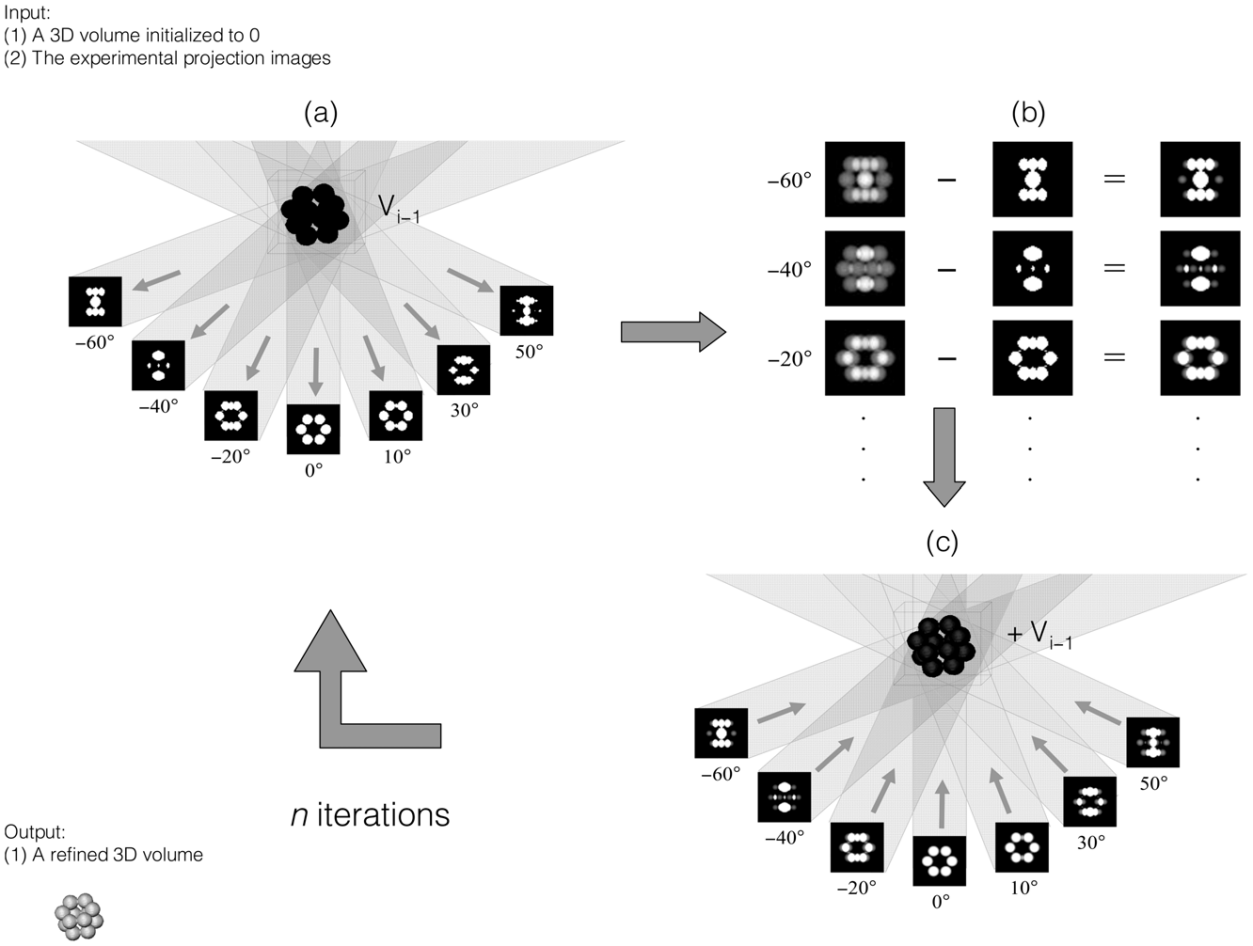
Three-dimensional reconstruction with SIRT. The reconstruction is progressively refined by minimizing the average error between the experimental and the calculated projections. (a) Calculation of projections from the volume at the current iteration. (b) Computation of the error with respect to the experimental projections. (c) Refinement of the volume by backprojection of the error. If 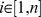 and represents the current iteration, then 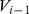 denotes the volume reconstructed in the previous iteration. 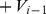 indicates that the volume generated in the previous iteration is taken into account to build the volume of the current iteration. In general, the volume is initialized to 0, that is, 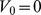.

**Figure 3:**
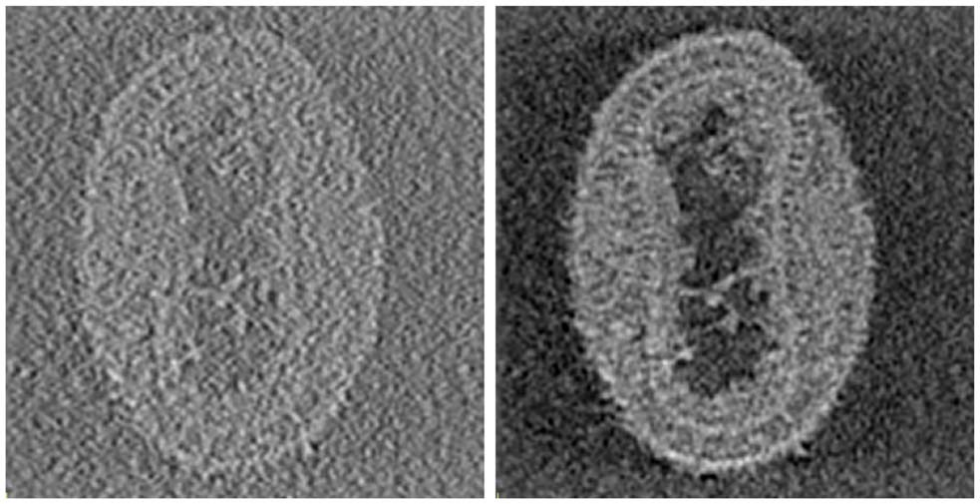
Three-dimensional reconstruction of Vaccinia virus. Tomogram obtained with WBP (left) and 30 iterations of SIRT (right). A 1.64 nm thick XY plane of the 3D reconstruction is shown. The tilt-series contained images in the range 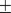 60 degrees at an interval of 2 degrees.

The reconstruction problem is then to obtain the 3D structure of the specimen from the set of projection images. Weighted back-projection (WBP) [44] is currently the standard algorithm in ET. WBP assumes that the projection images represent the amount of mass density encountered by the imaging electron beam. The method simply distributes the known specimen mass present in projection images evenly over computed backprojection rays (Figure 1 (b)). This way, the specimen mass is projected back into a reconstruction volume (i.e. backprojected). When this process is repeated for all the projection images in the tilt-series, backprojection rays from the different images intersect and reinforce each other at the points where mass is found in the original structure. Therefore, the 3D mass of the specimen is reconstructed from a series of 2D projection images. The backprojection process involves an implicit low-pass filtering that makes reconstructed volumes strongly blurred. In practice, in order to compensate the transfer function of the backprojection process, a previous high-pass filter (i.e. weighting) is applied to the projection images, hence the term “weighted backprojection”. This weighting is necessary to properly represent the high frequency information in the reconstruction [44]. For a detailed description of the method, refer to [44]. The relevance of WBP in ET mainly stems from its computational simplicity. Its disadvantage is the sensitivity to the conditions found in ET, namely the limited tilt angle and low SNR.

**Figure 4:**
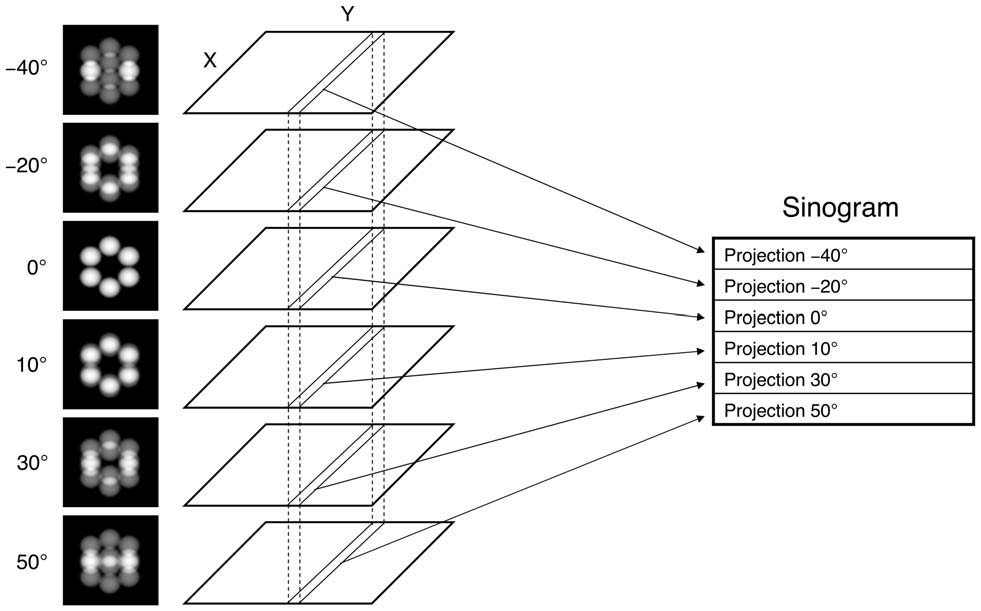
How a sinogram is built. All projection images are stacked and the 1D projections (or, simply, projections) that belong to the same slice (those between the vertical dotted lines) are grouped into a sinogram. This process is repeated for every slice. Therefore, there will be as many sinograms as slices.

There exist alternative real-space reconstruction algorithms that formulate the 3D reconstruction problem as a large system of linear equations to be solved by iterative methods [1]. These methods are more robust and overcome the limitations of WBP, though they may present a problem of potential overfitting [19]. In essence, these methods refine the volume progressively by minimizing the error between the experimental projection images and the equivalent projections calculated from the reconstructed volume. A very well accepted iterative method in the ET field is SIRT, which stands for Simultaneous Iterative Reconstruction Technique [45]. In every iteration of SIRT, (1) projections from the current volume are computed; (2) the error between the experimental projections and those computed from the volume is calculated; (3) the volume is refined by backprojection of the average error (Figure 2).

**Figure 5:**
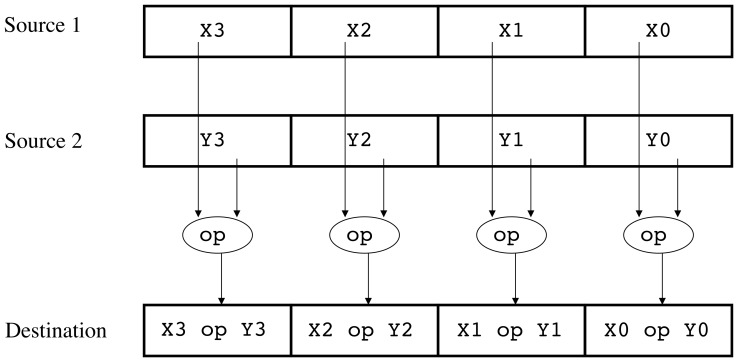
SIMD execution model. *Source 1* and *Source 2* are vector registers. Each one contains several data elements, which can be integer or real numbers. The same operation (*op*) is carried out between the two registers as indicated and the result is stored in another one (*Destination*).

For illustrative purposes, Figure 3 shows a comparison of the performance of WBP and SIRT on a dataset of Vaccinia virus [46]. The latter shows a dramatic improvement in contrast. The advantages of the SIRT reconstruction for interpretation of the structure are thus evident. Despite the great benefits of SIRT, its use has been limited by its computational demands. Approximately, every iteration takes twice the time of WBP, and a number of iterations in the range 20–50 is the standard, which makes it up to two orders of magnitude slower than WBP. The use of HPC techniques is therefore paramount to make SIRT competitive in terms of turnaround time.

**Figure 6:**
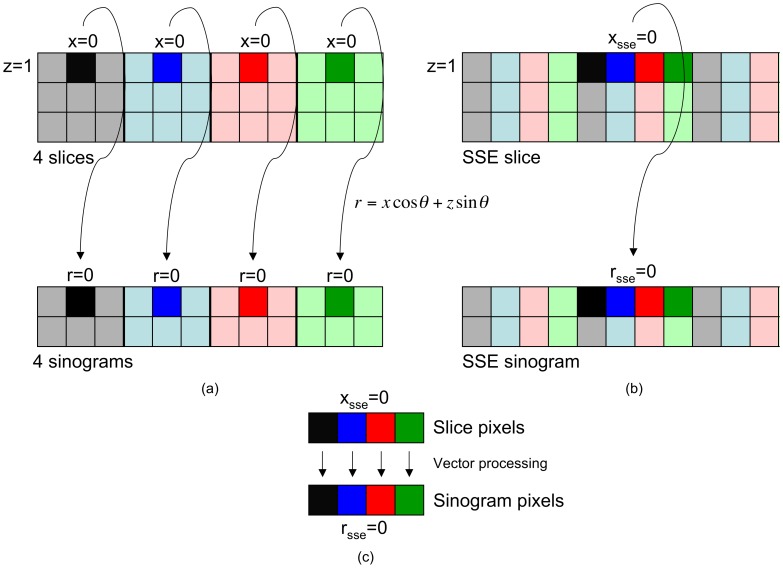
Arrangement of data for vector processing. Four different slices are sketched (coloured in grey, blue, red and green), and also the four sinograms associated with them. Every sinogram is composed of two projections. (a) Native data layout. (b) Data arrangement to take advantage of vector units. (c) Vector processing between data elements.

Assuming voxels as basis functions to represent the volume, the 3D reconstruction problem can then be decomposed into a set of independent two-dimensional (2D) reconstruction subproblems corresponding to the 2D slices perpendicular to the tilt axis [12] (see Figure 1 (a) where a slice is sketched). The reconstruction of a 2D slice is computed from the corresponding set of 1D projections (so-called sinogram, Figure 4), using the same algorithms but now working in 2D. The 3D volume is then obtained by stacking the reconstructed 2D slices. This decomposition has been extensively used for the development of efficient HPC approaches to this problem [12]–[14], [22], [26], [28], [29]. In ET, the tilt axis typically runs along the Y axis. Therefore, the 2D slices are in the XZ plane, which is the convention used hereinafter.

**Figure 7:**
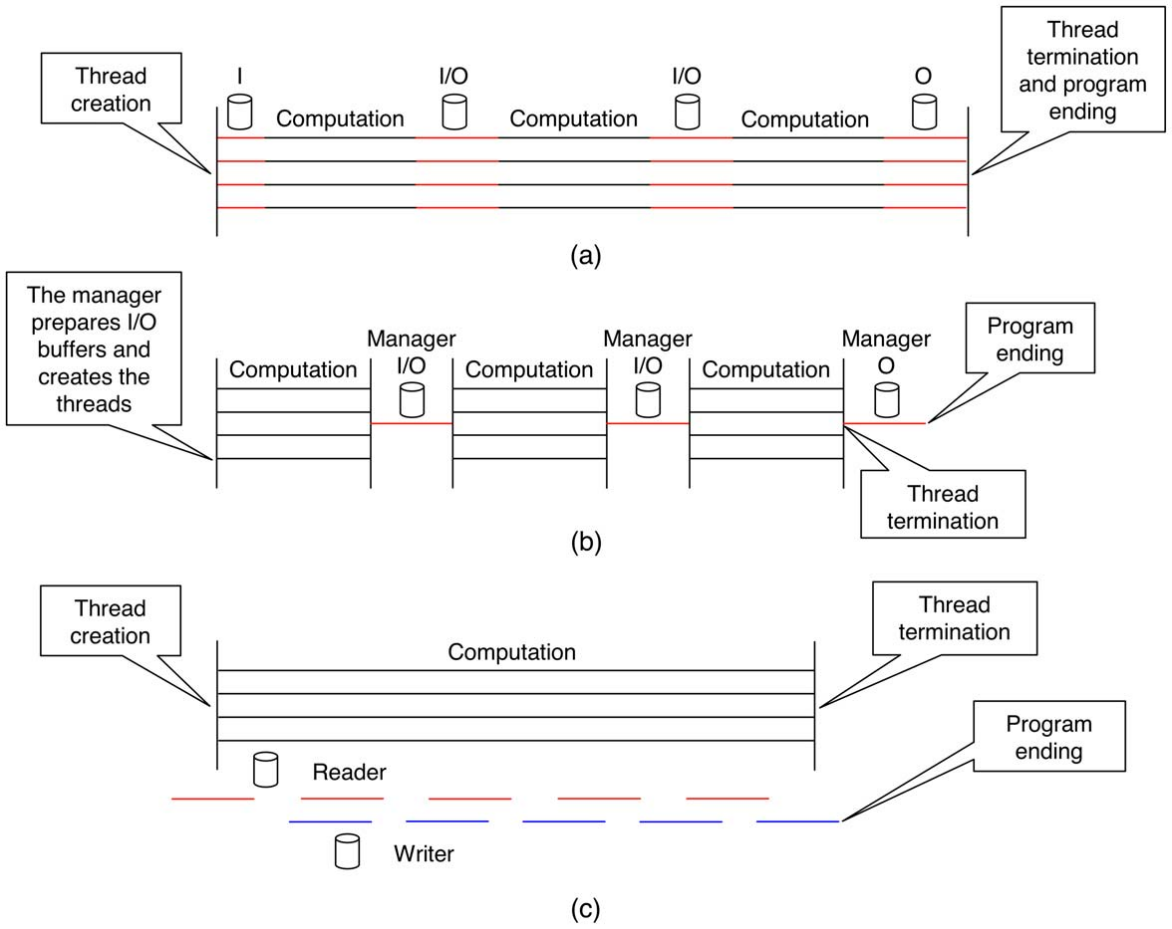
Schemes using multithreading. Each one represents four (working) threads as black parallel horizontal lines. ‘I’ stands for input (i.e. disk read), while ‘O’ means output (i.e. disk write). (a) is the static scheme. Note that every thread fills its input buffer before starting to reconstruct. (b) is the dynamic scheme. The manager is in charge of I/O operations. (c) is the dynamic with asynchronous I/O scheme. Here the manager is replaced by the so-called I/O threads (the reader and the writer). The reader needs to start shortly before the working threads in order to fill the input buffer. It finishes when there are no more sinograms to read. The writer begins when some slices have been reconstructed and finishes when it writes to disk the last pack of reconstructed slices.

The 2D tomographic reconstruction process by WBP and SIRT can be mathematically expressed in simple terms as the following formulae, respectively:
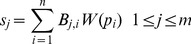
(1)

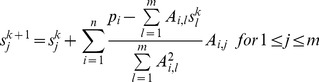
(2)where 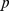 denotes the set of experimental 1D projections (i.e. the sinogram) and 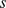 is the reconstructed slice, with size 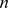 and 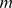 respectively. 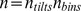, with 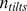 being the number of projection angles and 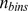 the number of projection values obtained for every projection angle, and 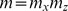, with 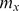 and 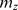 being the number of voxels in the 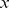 and 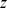 dimensions of the slice, respectively. 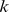 represents the iteration index in SIRT. 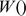 represents the high-pass filtering operation involved in WBP. The coefficient 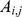 of the matrix 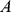 is a weighting factor representing the contribution of the voxel 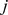 to the projection value 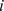, and its value only depends on the geometry of the projections. This matrix is sparse, i.e. many coefficients are zero since the contribution of every voxel is associated with a small subset of projection values. In particular, for a given tilt angle 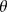, a voxel 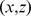 of the slice is projected to the point 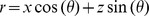 in the projection vector. The matrix 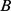 is the transpose of matrix 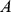.

**Figure 8:**
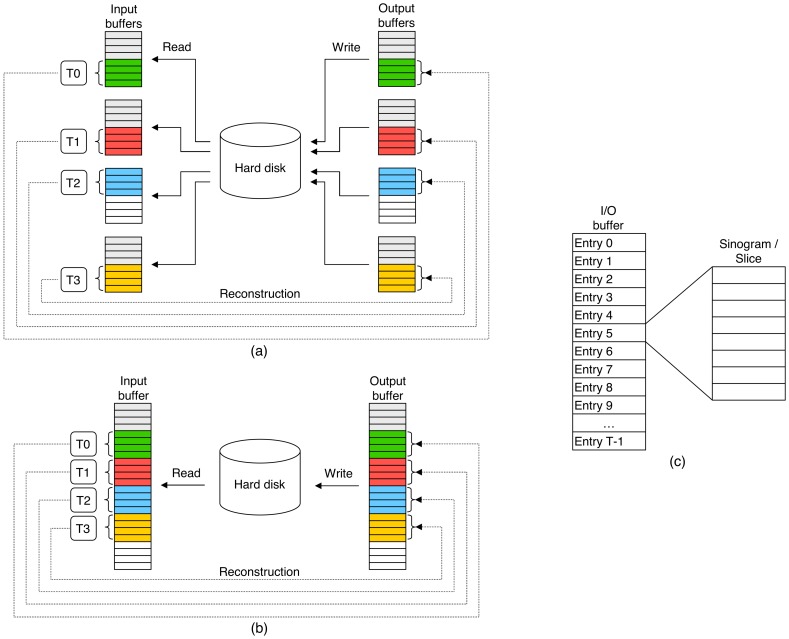
Workload distribution. (a) Static scheme. All threads (*T0, T1, T2, T3*) are allowed to perform disk I/O. Here I/O buffers are private and every thread has an identical pre-assigned amount of slices to reconstruct. (b) Dynamic schemes. Disk I/O is not carried out by threads anymore. There exist a shared input buffer where threads go to look for work. Once reconstructed, the slices are put in the shared output buffer. In (a) and (b) sinograms and slices are allotted to threads in slabs of four. Slabs in grey have been already processed, while those coloured (green, red, blue, orange) are being processed. The white ones have not been used yet. (c) An I/O buffer. If the buffer holds sinograms, it is called ‘input buffer’. On the other hand, if it keeps slices, it is called ‘output buffer’. The number of entries in an input buffer does not need to match the number of entries in an output buffer.

## Methods

In this section, our approach to fast tomographic reconstruction is presented. We disect the different optimizations applied to the reconstruction process, which can be divided into three main groups: (1) basic optimizations, (2) vector processing and, lastly, (3) multithreading and disk I/O enhancement. Figure S1 shows a flowchart of the optimization procedure.

**Table 1:** Reconstruction times.

	WBP			SIRT		
Optimization	512	1024	2048	512	1024	2048
Original	163.48	659.07	2658.91	–	–	–
Basic	26.30	106.25	424.15	1655.31	6622.12	26421.20
Speedup	6.22	6.20	6.27	–	–	–
SSE	7.45	30.72	124.42	447.44	1821.40	7323.16
Speedup	21.94 (3.53)	21.45 (3.46)	21.37 (3.41)	3.70	3.64	3.61
2T	3.80	15.62	62.95	223.88	912.10	3666.85
Speedup	43.02 (1.96)	42.19 (1.97)	42.24 (1.98)	7.39 (2.00)	7.26 (2.00)	7.20 (2.00)
4T	1.90	7.91	32.19	111.97	456.65	1843.53
Speedup	86.04 (3.92)	83.32 (3.88)	82.60 (3.86)	14.78 (4.00)	14.50 (3.99)	14.33 (3.97)
8T	0.99	4.21	15.90	56.78	239.25	1014.69
Speedup	165.13 (7.52)	156.55 (7.30)	167.23 (7.82)	29.15 (7.88)	27.68 (7.61)	26.04 (7.22)

Reconstruction times (in seconds) for WBP (left) and SIRT with 30 iterations (right). Speedups not in brackets are accumulated, while those in brackets are the contribution of individual optimizations. Some data were previously presented in [37].

**Figure 9:**
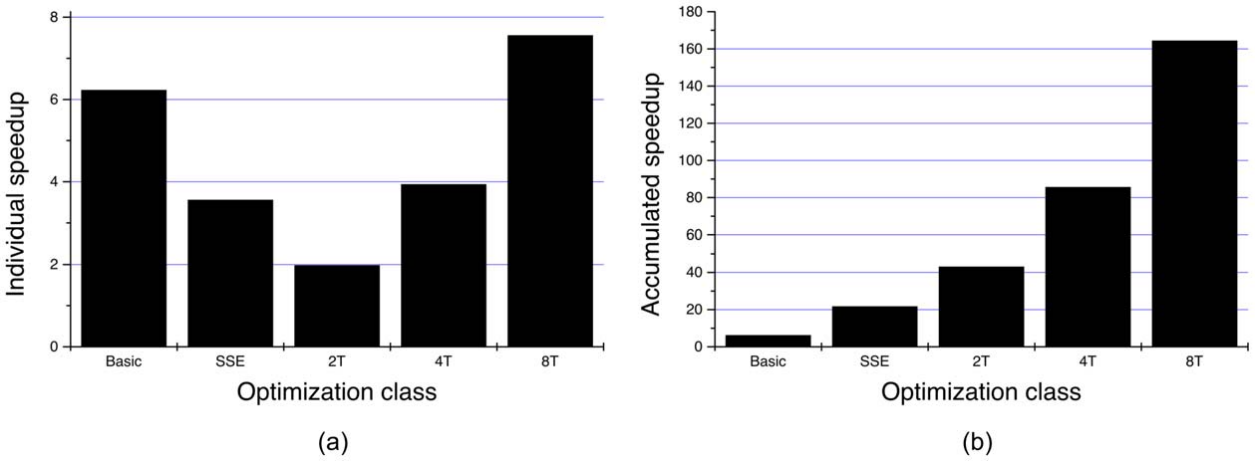
Speedup. Both WBP and SIRT have been taken into account. (a) Speedup provided by individual optimizations. Using the basic optimizations we reach a speedup slightly higher than 6x, with SSE instructions we are close to the theoretical 4x, and with two and four threads we can say that the speedup is linear with the number of cores. With eight threads it decreases a little. (b) Accumulated speedup. If we include basic optimizations and SSE instructions, the speedup is around 20x. When using two, four and eight threads, it rises above 40x, 80x and 160x, respectively.

### Basic Optimizations

Basic optimizations mission is to provide fast, sequential algorithms upon which parallel versions could be risen. Here we include a set of single-processor code optimization techniques that have been proved to improve the performance in scientific computing [47].

**Figure 10:**
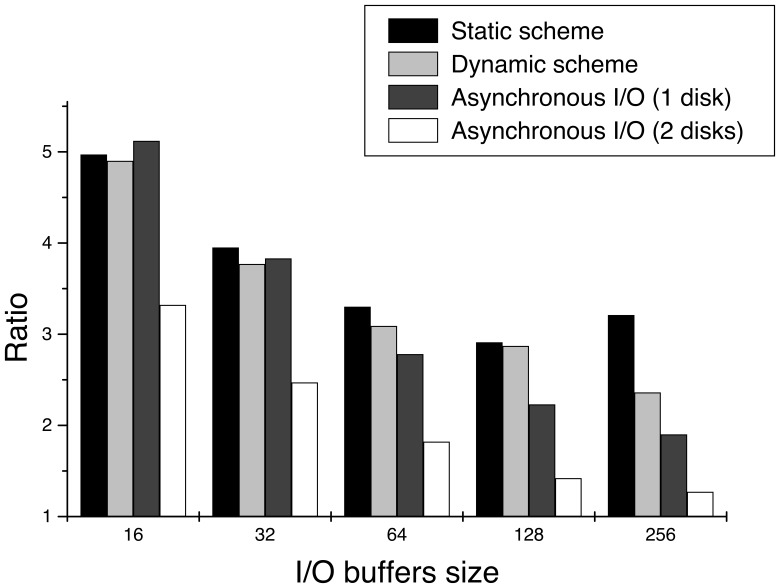
Ratio 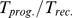 in WBP. The larger the I/O buffers, the lower the ratio. The asynchronous I/O is the scheme with the lowest ratio, particularly when using two hard disks. In this case, it is very close to 1 with buffers of size 128 or 256, which means that almost all the I/O is being overlapped. Note that the ratio of the dynamic scheme is similar to that obtained by the static one, except for 256. Though we have seen that the dynamic approach is better, here we are including smaller volumes (4 GB versus 8 GB) and the differences between this two strategies are mitigated.

**Figure 11:**
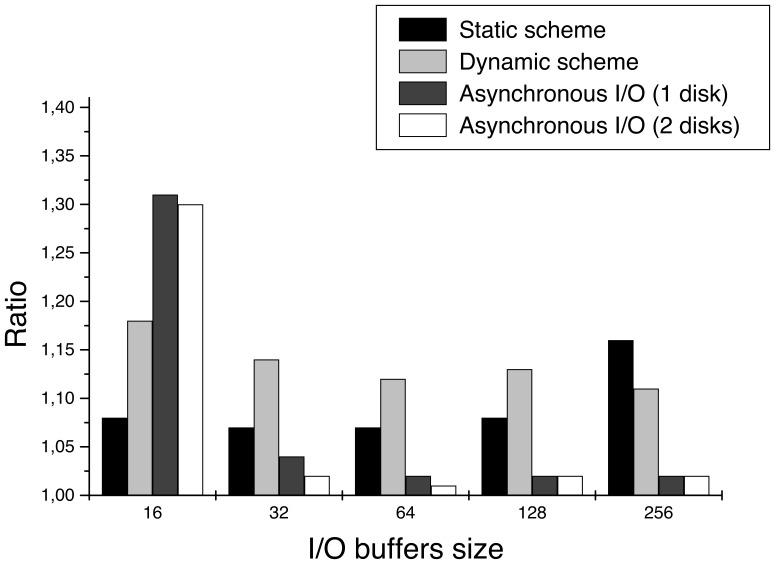
Ratio 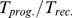 in SIRT. The asynchronous I/O is the best of the three schemes, but in SIRT there is not a significant difference between using one or two hard disks. It can also be observed that buffers as big as in WBP are not needed to obtain good ratios. The dynamic scheme behaves the worst since it has the highest ratios.

#### Efficient use of the cache memory

During the reconstruction process, both slices and sinograms are divided into blocks to make the most of processor cache. This procedure is very similar to the blocking technique widely used in scientific computing [47]. Its goal is to minimize the exchange of information with main memory by reusing the data kept in cache to a great extent. To this end, the data are split in small blocks that fit into cache memory and the code is reorganized so as to operate with a block as much as possible before proceeding with a different block. An optimal block size is selected automatically at runtime. It is typically set up as a portion (around 1/6) of the available cache memory.

**Table 2:** Load balancing in WBP.

	WBP					WBP with overload				
Scheme	*T*_prog._	T0	T1	T2	T3	*T*_prog._	T0	T1	T2	T3
Static scheme	49.95	512	512	512	512	301.64	512	512	512	512
Dynamic scheme	53.23	512	512	512	512	91.71	256	832	128	832
Asynchronous I/O (1 disk)	54.41	508	512	508	520	78.53	224	848	128	848

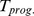
 (in seconds) and the number of slices reconstructed by each thread (T0, T1, T2, T3) are shown. The static scheme is deeply affected by the delay, while the dynamic approach responds well to the abnormal situation. When the asynchronous I/O is used, the best results are obtained because there are no synchronization points where the threads need to wait for each other.

**Table 3:** Load balancing in SIRT.

	SIRT					SIRT with overload				
Scheme	*T*_prog._	T0	T1	T2	T3	*T*_prog._	T0	T1	T2	T3
Static scheme	472.09	512	512	512	512	712.76	512	512	512	512
Dynamic scheme	477.49	512	512	512	512	572.02	448	640	384	576
Asynchronous I/O (1 disk)	473.28	516	504	504	524	543.57	464	600	384	600

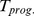
 (in seconds) and the number of slices reconstructed by each thread (T0, T1, T2, T3) are shown. The static scheme is deeply affected by the delay, while the dynamic approach responds well to the abnormal situation. When the asynchronous I/O is used, the best results are obtained because there are no synchronization points where the threads need to wait for each other.

#### Projection symmetry

This optimization takes advantage of the symmetry existing in the projection of a slice: if a point 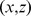 of the slice is projected to a point 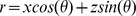 in the projection corresponding to the tilt angle 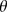, it is easy to see that the point 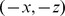 of the slice is then projected to 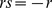 in that projection (note that 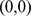 is the centre of the slice). Therefore, for a given tilt angle 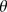, there is only need to compute the point 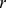 in the projection for half of the points 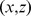 in the slice, hence obtaining a gain in speed. To further increase cache efficiency (see previous section), in this work symmetric points are put together in the data structures.

**Table 4:** Comparison CPU vs. GPU (backprojection).

GPU/CPU	60	90	120	VV2K
GTX 295 [27]	6.19	7.95	9.54	26.05
E5405 (8T)	7.96	10.78	12.11	32.82
Q9550 (4T)	10.83	14.46	18.24	42.75

We are comparing our implementation of backprojection with [27], where a NVIDIA GTX 295 was employed. Although the GPU performs better, it is important to note that the differences are small, specially when the E5405 is used.

**Table 5:** Comparison CPU vs. GPU (SIRT, 1 iteration).

GPU/CPU	Dataset A	Dataset B	Dataset C
GTX 280 [29]	2.10	10.66	58.47
Tesla C1060 [28]	0.53	4.80	51.28
GTX 285 [28]	0.46	3.90	42.32
Tesla C2050 [28]	0.57	3.70	37.91
Q9550 (4T)	0.48	4.03	32.17
E5405 (8T)	0.35	2.94	24.55

All the GPUs are from NVIDIA. The Q9550 clearly outperforms the GTX 280 and it gives times of the same order as those obtained with the other GPUs. The E5405 is the absolute winner of the benchmark. Some data were previously presented in [37].

**Table 6:** Comparison with IMOD.

	WBP		SIRT	
Implementation	1024	2048	1024	2048
IMOD	59.66	218.82	462.48	1800.46
Our approach - 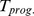 (1 disk)	23.17	85.58	60.16	249.24
Our approach - 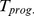 (2 disks)	14.55	49.48	59.03	248.43
Our approach - *T_rec._*	6.91	28.50	55.82	243.64

Wall time (in seconds) spent by IMOD and our approach (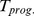) to reconstruct the 1024 and 2048 datasets with WBP and SIRT (5 iterations). 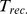 denotes the time spent solely in the reconstruction, i.e. without I/O, by our approach.

#### Other optimizations

A wide spectrum of single core optimizations has been applied to further accelerate the code [47]. Among them, we highlight (1) an instruction level parallelism increase, (2) pre-calculation of data that are extensively used during the reconstruction process (e.g. sines, cosines, rays, limits for projections), (3) inlining of functions, (4) replacement of power of two divisions and multiplications by shifts, (5) replacement of some divisions by multiplications, (6) loop unrolling and (7) conditionals removal.

### Vector Processing

Today's processors offer the programmer a set of vector units, which need to be explicitly programmed if maximum exploitation is sought. We have used the SSE (Streaming SIMD Extensions) instructions present in all current processors from Intel and AMD to further accelerate the tomographic reconstruction. These instructions are able to carry out the same operation (e.g. a division) over several data elements in just one step (Figure 5). Among the data types they can deal with, we highlight single-precision floating point numbers (32 bits), since in the ET field the points of a tomogram are usually in this format. SSE registers are 128-bit long, which means they can hold up to four floats.

Modern compilers, such as the Intel compiler or the GNU compiler (gcc), are able to perform an automatic vectorization of the source code, but in practice this is restricted to rather simple loops. For that reason, it becomes imperative to hand-write the code to really take advantage of vector units. We have selected the C language and compiler intrinsics for our algorithms.

Figure 6 depicts our vectorized approach to tomographic reconstruction, whose purpose is to reconstruct four slices simultaneously. The key lies in the fact that voxels belonging to different slices, but located at the same coordinate, are linked with the same value of 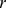 when the formula 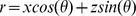 is applied, as the formula only depends on the coordinate 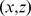 and the angle 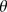. For example, by choosing 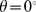, the black pixel in the grey slice located at 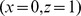 (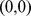 is the centre) will be linked with the black pixel in the grey sinogram located at 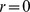 (Figure 6 (a)). If we focus on the other slices, we will realize this is also true for their pixels placed at 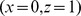.

Operations carried out between the two black pixels are exactly the same that the ones performed between the two dark blue pixels, the two dark red pixels and the two dark green pixels. This leaves the door open for processing them through vector instructions. Nonetheless, it is not possible because of the current data layout, i.e. pixels we are interested in are placed in non-contiguous memory locations. If we arrange (pack) the data as presented in Figure 6 (b), those pixels will be together. As it can be noted, the first column of each slice is put firstly, then the second ones come, then the third ones and so on. Now it is feasible to read in parallel the four pixels of the slices and the four pixels of the sinograms. They will be loaded into vector registers and will be operated by means of vector instructions (Figure 6 (c)). Finally, the result will be stored into the corresponding memory location.

Now four pixels are being reconstructed at the same time, each one belonging to a different slice. In contrast, the sequential versions of WBP and SIRT are obliged to repeat the process for each pixel individually. Once reconstructed, the SSE slice has to be unpacked to get the four slices (i.e. the previous data arrangement is undone). These operations (pack and unpack) take an extra time, but it is negligible if compared to that required by the reconstruction algorithms.

For the weighting process involved in WBP, the FFTW (the Fastest Fourier Transform in the West) library has been employed [48]. This library takes advantage of SSE instructions internally and, therefore, vectorization is used throughout the WBP procedure.

### Multithreading and Disk I/O Optimization

Current commodity processors are shipped with several cores (e.g. 2 to 8), and modern computers may have several of such processors. If full exploitation of those computing cores is intended, they must be explicitly programmed. To that end, we have used the Pthreads library, by which it is possible to split an application into threads making each one completing a piece of the total workload. As we have already mentioned, a 3D volume (or tomogram) can be divided into 2D slices that can be reconstructed independently. In our multithreaded approach, we create as many threads as available cores, and the slices are distributed among those threads. Every one runs a reconstruction algorithm (WBP or SIRT) previously optimized using the basic optimizations and vector processing. We have developed three schemes (Figure 7), which differ in the workload distribution strategy and the way disk I/O is performed.

Workload distribution can be static or dynamic. In the static case (Figure 8 (a)), the slices are equally allotted to the threads. Once the distribution is carried out, it cannot be undone, which makes load balancing absent. In the dynamic case (Figure 8 (b)), the 3D volume is seen as a warehouse or pool of slices where the threads autonomously go to look for work. This fact makes possible load balancing as faster threads will get more slices to process. Slices are delivered in slabs of four to be reconstructed through vector processing.

Disk I/O is performed using custom-made buffers. A buffer is just a structured memory region that temporarily stores sinograms (input buffer) or slices (output buffer) (Figure 8 (c)). During the reconstruction process, sinograms are not read from disk one by one. Instead, a set of them is loaded into the input buffer, which is in charge of feeding the threads. Similarly, slices are not immediately written to disk when reconstructed, but temporarily stored in the output buffer. Depending on the scheme, the input buffer will be reloaded when it is entirely empty or it will be inspected from time to time to replace those sinograms already processed. Likewise, the output buffer will be dumped to disk when completely filled or it will be checked periodically to physically write already reconstructed slices, thus making space for new ones. The goal of I/O buffers is to decrease the number of disk accesses, hence reducing the time I/O operations need.

#### First scheme: static load distribution

In this approach (Figure 7 (a) and Figure 8 (a)), all the threads receive the same amount of slices to reconstruct. For example, if there are 4 threads and 64 slices, each one will receive 16 slices. If the number of slices is not divisible by the number of threads, then the fairest distribution is carried out. For instance, assuming 3 threads and 64 slices, two threads will receive 20 slices and the remaining one, 24 (note that the minimum workload unit is four because of vector processing). Here the threads are completely independent and so, there are no communication latencies. The price paid for independence is a fixed workload distribution that cannot be undone at runtime and makes load balancing impracticable. Independence also forces to duplicate resources and thus, I/O buffers are private, that is, each thread has its own input and output buffer.

The scheme works as follows. Every thread fills its input buffer by reading a set of sinograms from disk. Then, the slices are stored in the output buffer as they are reconstructed. When a thread detects its input buffer is empty, it will reload the buffer with new sinograms. In a like manner, when its output buffer is full, the thread will dump the reconstructed slices to disk. This process is repeated until all the threads have their allotted slices reconstructed.

Apart from the absence of load balancing, this approach has several disadvantages. One is a high memory consumption. Basically, memory usage depends on two factors: the size of I/O buffers and the reconstruction dimensions. For example, assuming I/O buffers with 64 entries and 140 projection images of 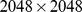 pixels to yield slices of the same size (every pixel is a 4-byte single-precision float), an input buffer would require 70 MB, while an output buffer would need 1 GB. Since I/O buffers are replicated as many times as threads, these numbers have to be multiplied by the thread count. Nowadays it is common to find processors with four, six or even eight cores, not to mention that many are hyper-threaded and that some computers are equipped with more than one processor. If we exploit all the cores, the system could easily run out of memory.

Another disadvantage of this approach is that all threads are allowed to perform I/O, which can result in an inefficient disk access. When a read operation is ordered, more data than requested are stored in the disk cache. This cache has a limited size, and if more than one thread is reading from disk, data inside the cache will be constantly overwritten. On the other hand, we could be forcing a constant hard disk head-positioning as we would not be reading or writing sequentially.

#### Second scheme: dynamic load distribution

This strategy (Figure 7 (b) and Figure 8 (b)) aims to solve the problems the static scheduling has, that is, absence of load balancing, high memory consumption and inefficient disk access. The main difference is that now I/O buffers are not replicated: there only exist one input buffer and one output buffer, which are shared among all threads. Because of this, the amount of required memory does not depend on the number of threads anymore. In contrast, it is mandatory a mechanism to control the access to I/O buffers, or more than one thread could reconstruct the same slab of slices. Thus, accessing buffers becomes a critical section and only one thread is granted the permission.

A thread inside the critical section should leave it as soon as possible or performance will degrade to a large extent. In our case, a thread just asks for a slab of slices not yet reconstructed and it receives a pointer to the corresponding sinograms and a pointer where the slices have to be stored. Then, it exits. These pointers point to the input and output buffer, respectively.

In this scheme we have an additional thread called the manager. The manager creates the working threads (those which reconstruct slices) and prepares the I/O buffers, i.e. fills the input buffer with sinograms and makes ready the output buffer so that reconstructed slices can be stored inside. After that, the working threads start running and the manager stays listening. When a thread detects that the input buffer is empty or the output buffer is full, it notifies the manager and goes to sleep. Then, the manager reloads the input buffer with new sinograms and dumps reconstructed slices to disk, hence making new space in the output buffer. Once I/O buffers are again ready, the manager wakes up the working threads. If there were no more slices to reconstruct, the working threads would be informed and terminated.

Now a slow thread will not delay the reconstruction as much as in the static approach: it will simply request less slices, which results in an implicit and dynamic load balancing. However, it is possible for a slow thread be a burden for the others. This can happen when there are no more sinograms to process in the input buffer or there is no space left in the output buffer and a slow thread is reconstructing the last slab of slices it took. The other threads cannot continue because I/O buffers are not ready and the manager cannot prepare them because the slow thread has not finished yet.

I/O operations are now put in order since the manager is the only one allowed to perform them. This should correct the inefficiencies discussed in the previous section, but this rises a new matter: during the time the manager is doing I/O, the working threads are sleeping and therefore no slices are being reconstructed.

#### Third scheme: dynamic load distribution with asynchronous I/O

In this last scheme (Figure 7 (c) and Figure 8 (b)), the dynamic load distribution is kept thanks to the shared I/O buffers, but the manager is replaced by two threads in charge of performing I/O operations. One is called the reader and it is responsible for filling the input buffer with new sinograms as working threads process them. The other is the writer, which dumps reconstructed slices to disk. Since I/O operations are uncoupled (i.e. they are carried out by separated threads), they can run in parallel if two different hard disks are used, one for reading and another for writing.

I/O threads are sleeping most of the time. They wake up from time to time to check the state of I/O buffers and run concurrently with working threads. If there are sinograms already processed in the input buffer, they will be replaced with new ones. If there are reconstructed slices in the output buffer, they will be dumped to disk and the space will become available again. Due to this behaviour, I/O is asynchronous and overlaps with computation, that is, the working threads do not need to wait for I/O operations to complete as they can reconstruct slices while I/O takes place.

It is not always possible to overlap all the I/O, particularly when it takes more time than computation. For instance, the output buffer can have no free entries to accommodate new reconstructed slices if working threads are so fast to completely fill it before the writer is able to dump some slices. In these cases, working threads will go to sleep while I/O buffers become ready again.

Using this scheme, a slow thread cannot delay the others because there are no synchronization points where the working threads have to wait for each other, something that could happen in the previous approach. Of course, a slow thread will always delay the execution, but here the effect is mitigated regarding the other schemes.

## Results and Discussion

We have grouped our experimental results into several sections. The first presents reconstruction times, the second analyses disk I/O times, the third focuses on load balancing, the fourth compares our implementation of WBP and SIRT with some others written for GPUs and, finally a comparison with a standard package in the ET field is carried out in the last one. All the experiments were carried out on the Linux operating system and the Intel C/C++ compiler was used to compile our algorithms.

### Reconstruction Times

We have evaluated the performance of our reconstruction algorithms on a server-based computer equipped with two quad-core Intel Xeon processors E5405 at 2 GHz (each one with 12 MB of L2 cache) and 16 GB of RAM. Three different volumes were reconstructed: 512
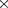
512
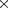
256, which is typical of real-time environments, 1024
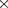
1024
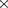
256, which is currently a standard volume, and 2048
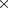
2048
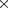
256, which will be adopted as the standard in the near future. These volumes were derived from datasets with projection images of size 512
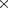
512, 1024
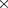
1024 and 2048
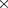
2048, respectively. Each dataset was composed of 140 projection images that were taken in the tilt range 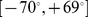 at intervals of 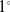.

Table 1 shows the reconstruction times obtained for WBP (left) and SIRT with 30 iterations (right). *512*, *1024* and *2048* represent the volumes 512
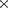
512
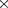
256, 1024
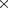
1024
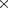
256 and 2048
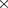
2048
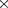
256, respectively. *Original* denotes the original version of WBP, that is, the one used as a starting point and not optimized. In the case of SIRT, we did not have an original version and, thus, this algorithm was written directly using the basic optimizations. *Basic* refers to the times achieved when only the basic optimizations were taken into account, while *SSE* means that vectorization was also included. In the previous cases, a single core was employed to run the algorithms. *2T*, *4T* and *8T* indicate that two, four and eight threads (one per core) were utilised, respectively. Every experiment was launched five times, and the average computation time was then calculated and expressed in seconds. In WBP, the speedups not in brackets were determined regarding the original version (for which automatic vectorization was enabled) and, in SIRT, regarding the basic optimizations. Those in brackets give the acceleration factor provided solely by the corresponding optimization.

With regard to WBP (Table 1 (left)), a speedup around 6x is achieved using the basic optimizations, no matter the volume reconstructed. If we focus on SSE instructions, we see that the overall acceleration factor is very close to 3.5x, which is great taking into account that the theoretical maximum is 4x. If we join these two optimizations, we obtain an accumulated speedup higher than 20x that rises to 40x, 80x and 160x when using two, four and eight threads, respectively. As far as the use of multithreading is concerned, it approaches linear speedup with the number of threads, though with eight threads it decreases slightly.

With regard to SIRT (Table 1 (right)), the acceleration factor given by the SSE instructions is again quite good (around 3.6x), actually somewhat better than in WBP. The speedup provided by two and four threads is once more linear with the number of cores (2x and 4x, respectively). With eight threads, it decreases a little, but keeps staying close to the maximum (always greater than 7.2x). Although SIRT was written directly using the basic optimizations, it is based on WBP and, thus, it is reasonable to think that a similar speedup would be obtained with those optimizations over a hypothetical original version. This fact would be reinforced by the other optimizations (*SSE*, *2T*, *4T* and *8T*) as the acceleration factors achieved in both WBP and SIRT have the same order of magnitude. This way, if we multiply the accumulated speedups of SIRT by 6, we get a global speedup around 20x, 40x, 80x and 160x for vector processing, two, four and eight threads, respectively.

Figure 9 (a) presents the acceleration factor each optimization gives by itself. To build this graph, the speedups in brackets shown in Table 1 were used. For each optimization, the mean was calculated taking into account the speedups related to both WBP and SIRT. Similarly, Figure 9 (b) exhibits the accumulated acceleration factor as we add optimizations to the reconstruction algorithms. In this case, the speedups not in brackets were used and the mean was again computed. For SIRT, we considered that the gain over a hypothetical original version was 6x and, thus, we multiplied the corresponding speedups by 6.

The final processing time that has been obtained for both methods is remarkable. The calculation of the tomogram *512* with WBP on 8 cores is ready in just 1 second, which definitely enables real-time reconstruction. On the other hand, the tomograms *1024* and *2048* reconstructed with SIRT using 8 cores are available in just 4 and 17 minutes, respectively. These turn out to be reasonable reconstruction times from the user's point of view. If an unoptimized SIRT code was used, the processing time for this method would be prohibitive, which traditionally has precluded its extensive application in the field.

### I/O Times

In the previous section, we have analysed the reconstruction times, but nothing has been said about the time required to read datasets from disk and write volumes to disk. When huge volumes are reconstructed, disk I/O can consume a significant portion of the program time (i.e. wall time) and can even be higher than the reconstruction one. Here we compare the three multithreaded strategies discussed earlier (i.e. static, dynamic and dynamic with asynchronous I/O) and examine how our optimizations related to I/O behave. To that end, we have reconstructed a volume of 2048
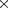
2048
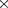
512 voxels that totals 8 GB. We wanted a big volume to exhibit the differences among strategies and we selected this one. It was derived from a dataset composed of 140 projection images, each with a size of 2048
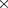
2048 pixels. This dataset needs 560 MB of hard disk space (in this dataset every pixel takes up a byte). The computer chosen for the experiments was equipped with an Intel Core 2 Quad processor (four cores) Q9550 at 2.83GHz and 8 GB of RAM. It had also two SATA hard disks at 7200 rpm. Every experiment was run five times and then, the mean (in seconds) was calculated. WBP and SIRT were launched with the best configuration, that is, basic optimizations plus vector processing plus one thread per core. Only 5 iterations of SIRT were used as they were adequate for our purposes. In order not to distort the experiments, the disk cache was emptied between runs.

Table S1 shows the results for WBP (left) and SIRT (right). Five different sizes for I/O buffers have been used: 16, 32, 64, 128 and 256. For example, 64 means that the input buffer could hold 64 sinograms and the output buffer, 64 slices. 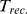 represents the reconstruction time, 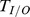 stands for disk I/O time and 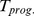 signifies program time. Usually, 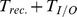 will approach 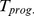. In the case of asynchronous I/O, 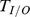 indicates the amount of I/O that could not be overlapped. *Mem.* denotes the amount of RAM memory (in gigabytes) consumed by the reconstruction.

In general, as we increase the buffer size, 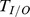 decreases, no matter the reconstruction algorithm or the multithreaded strategy. There is just one case where this is not true: when the size is 256 and the static scheme is used. Probably, the reason is that the memory required (more than 5 GB) is too high. Although the computer we chose had 8 GB of RAM, we have to take into account that the memory must be shared between our data structures and those used by the operating system. So, it is very likely that our I/O buffers do not fit in RAM and, thus, swapping is occurring. On the other hand, the static strategy is the one that needs more memory throughout all the experiments.

With regard to WBP (Table S1 (left)), we can observe that I/O is very heavy since 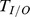 is consistently higher than 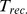, except when the asynchronous I/O scheme is used with two hard disks. The static scheme behaves the worst as 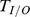 is always greater than 100 seconds while the others can approach 60 seconds, not to mention that asynchronous I/O with two hard disks gives I/O times just slightly above 10 seconds. As it can be seen, small sizes for I/O buffers, particularly 16 and 32, should be avoided regardless of the scheme. 64 seems enough for the static approach, while 128 is a good trade-off between memory consumed and I/O time in the case of the dynamic ones. In summary, the static strategy provides the poorest results. The dynamic and the dynamic with asynchronous I/O schemes are similar, but the latter is the best of the two, specially when two hard disks are used.

With regard to SIRT (Table S1 (right)), the I/O behaviour is different from that observed in WBP. The main difference is that I/O times are much shorter now than before, even though we are reconstructing the same volume and, therefore, we are reading and writing the same amount of data. The reason lays in the way I/O is performed by the operating system (Linux for us). When we order a ‘write to disk’ operation, the data we want to write (slices in our case) are not physically written immediately. Instead, they are temporarily stored in the disk cache implemented by Linux. When Linux deems appropriate, it dumps to disk the content of the cache. As SIRT has a towering computational load, dumping the cache can take place while new slices are reconstructed. In contrast, WBP is very fast and quickly fills the disk cache, forcing Linux to carry out a physical write. So, a ‘write to disk’ operation (which is what we can measure) usually means ‘write to disk cache’ in SIRT, while often signifies ‘write to disk cache and perform a physical write’ in WBP. Of course, the latter is much more expensive in terms of time. As a consequence, to perform an efficient disk access, I/O buffers can be smaller in SIRT (we can state that 64 is a good choice, no matter the strategy employed) and there is no need to use two hard disks in the asynchronous I/O scheme.

If the dynamic scheme is used, it can be observed in SIRT that 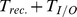 does not approximate to 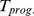. This is caused by the dependency between threads that exists when I/O buffers need to be prepared by the manager. As threads have to wait for each other in this synchronization point, the waiting times increase the program time. This effect is also present in WBP, but the waiting times are imperceptible since this algorithm is much faster. The problem is completely solved by the asynchronous I/O. In summary, now the dynamic scheme behaves the worst, then the static one comes and the winner is again the approach that uses asynchronous I/O.

Figure 10 and Figure 11 shows the ratio 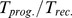 for WBP and SIRT, respectively. The closer the ratio is to one, the closer the reconstruction time is to the program time and, thus, the lighter the disk I/O is. This way, if the ratio equals to 1, then 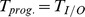, which is the main aim of the I/O optimizations presented in this work. Two more volumes were taken into account to build these graphs (1024
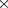
1024
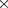
1024 and 2048
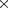
2048
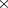
256, each one taking up 4 GB), although we have not included the tables with the results here.

### Load Balancing

Here we show the results related to load balancing. The computer and the volume chosen for the experiments were the same used in the previous section (I/O times). That volume (2048
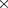
2048
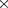
512 with 140 angles) was selected because it requires considerable processing time and so, it would highlight the differences among strategies. Again, 5 iterations of SIRT were picked as this amount was enough for the tests carried out here. Five runs were done of every experiment and the average time (in seconds) was then calculated. Both WBP and SIRT were launched with the best configuration, that is, basic optimizations plus vector processing plus four threads (one per core). I/O times were faded by writing the volume to/*dev/null* in order to focus on the reconstruction time.

To simulate a situation where the system is overloaded, we artificially delayed the threads 0 and 2. This delay consists of calling the function *sleep()* when those threads proceed to reconstruct a slab of slices. Each time this happens, thread 0 (*T0*) is paused one second and thread 2 (*T2*), two seconds. Table 2 and Table 3 show the results for WBP and SIRT, respectively. Each one is composed of two parts. The one to the left presents the results of a normal execution, while the one to the right exhibits the results of an overloaded execution. In addition to the execution time 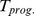 (in seconds), the number of slices each thread reconstructs is shown. As it can be noted, the static distribution is deeply affected by the delay since the workload distribution is fixed and cannot be undone at runtime. In contrast, the dynamic scheduling adapts well to the abnormal situation, employing much less time to perform the same reconstruction. Nonetheless, because of the dependency between fast and slow threads that exists in this scheme when I/O buffers needs to be prepared by the manager, asynchronous I/O becomes the winner strategy, either in WBP or SIRT. On the other hand, it is observed that the delayed threads (*T0* and *T2*) reconstruct less slices when using any of the dynamic schemes, that is, a load balancing is carried out. On the contrary, with the static approach all the threads process the same amount of slices.

When the scheme with asynchronous I/O is used and the system is not overloaded, it can be seen that some threads reconstruct some more slices than others, no matter the reconstruction algorithm. This is a normal behaviour which is just the result of a load balancing. Although we do not force a delay here, there can exist threads faster than others, for example, because a core can be shared by one of our threads and a process that belongs to the operating system in a certain moment. This could also happen in the dynamic scheme, but in this case fast threads must wait for the slow ones when I/O buffers need to be prepared. Therefore, the advantage that fast threads have is dropped.

### Comparison with GPUs

In the last few years, GPUs have shaken up the HPC field because of their tremendous computing power at an incomparable performance-to-cost ratio [23]. The ET community has rapidly adopted them and a number of GPU approaches for fast tomographic reconstruction have been proposed [24]–[29]. Here we compare our optimized multicore implementations of WBP and SIRT with the fastest GPU implementations reported thus far [27]–[29]. To facilitate the comparison, the same datasets as in [27]–[29] were used. The computers where we ran the experiments were the Q9550 and the E5405, whose characteristics have been already shown in previous sections. Table 4 presents the results for WBP, while Table 5 shows the ones obtained in SIRT. Again, the processing times (in seconds) are the mean of five runs. Only one iteration was selected for SIRT. For a fair comparison, we only took into account the reconstruction times, hence ignoring I/O times. Both WBP and SIRT were launched with the best configuration, that is, basic optimizations plus vector processing plus one thread per core.

In the case of WBP (Table 4), four different datasets were picked. Three of them were composed of (60, 90 and 120, respectively) 1024
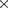
1024 projection images. All these datasets yielded volumes with dimension 1024
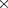
1024
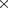
1024 voxels. The fourth dataset had 61 projection images of size 2048
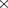
2048 (VV2K) and was used to generate a volume whose size was 2048
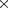
2048
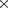
960 voxels.

In the case of SIRT (Table 5), three datasets were selected. They comprised 61 projection images of size 356
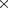
506 (*Dataset A*), 712
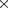
1012 (*Dataset B*) and 1424
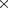
2024 (*Dataset C*), respectively. These datasets yielded volumes with dimensions 356
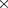
506
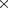
148, 712
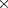
1012
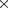
296 and 1424
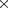
2024
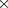
591 voxels, respectively.

In general, the performance of our WBP is very close to that published in [27]. If we focus on SIRT, our implementation is faster than those appeared in [28] and [29].

### Comparison with a Standard Package

In this section, we compare our approach with a standard software package in the field of electron tomography: IMOD [49] (http://bio3d.colorado.edu/imod). It is equipped with parallel implementations of WBP and SIRT. Essentially, they split the volume to be computed into chunks, which are subsequently reconstructed in parallel and finally reassembled to yield the definite tomogram.

In this comparison, we focused on the turnaround time, i.e. the total time that includes that used for reading input datasets and writing the results. The experiments were run on the computer based on Intel Core 2 Quad processor Q9550 used previously, which had two SATA hard disks at 7200 rpm. The parallel implementations in IMOD were used to exploit the four cores available in the computer. In our approach, the best configuration for processing (i.e. basic optimizations, vector processing, one thread per core, dynamic load balancing with asynchronous I/O) was set. The I/O buffers were configured to 128 entries and the possibility of taking advantage of the two hard disks was also exploited.

The experiments consisted of reconstructing volumes of 1024
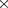
1024
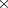
256 and 2048
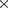
2048
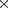
256 from 140 images of 1024
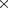
1024 and 2048
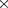
2048, respectively, with WBP and SIRT. In the particular case of SIRT, only 5 iterations were employed for this evaluation. In order to obtain fair time measurements and avoid distortion, the disk cache was emptied between runs.

The results of these experiments are summarized in Table 6. They clearly show that our approach outperforms IMOD. In the case of WBP, ours is faster than IMOD by a factor ranging from around 2.55 to 4.25, depending on whether one or two disks are used, respectively. In the case of SIRT, our approach achieves a remarkable speedup factor of 7.5 with regard to IMOD. As discussed previously, the influence of using one or two disks in SIRT is negligible. It is important to note that the parallel implementations available in IMOD rely heavily on I/O operations, which severely penalizes the performance. The good performance of our implementation stems not only from the optimizations at the basic, vectorization and multithreading levels, but also from the improvements to minimize I/O latencies. This is confirmed by the difference between the reconstruction time (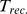) and the actual program time (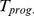) in Table 6, especially in SIRT.

### Conclusions

In this work, we have presented a detailed description and evaluation of a fast approach to tomographic reconstruction on multicore computers. We have developed highly optimized implementations of the algorithms WBP and SIRT. Different kinds of optimizations have been applied, which we have organised into three categories: (1) basic optimizations to build fast, sequential algorithms that could be used as a point of departure for parallel versions, (2) vector processing to take advantage of the vector capabilities of modern processors and (3) multithreading to capitalize on the various cores provided by multicore computers. Thanks to all these improvements, speedups of up to 160x have been reached. As a consequence, standard volumes can be reconstructed in a few seconds using WBP and several minutes through SIRT. This makes our algorithms competitive with current GPU solutions and suitable for real-time tomographic environments.

Apart from enhancing the reconstruction time, this work has also focused on two other topics: how to access the hard disk efficiently and how to balance the workload among the available cores. Regarding the first matter, we have proposed the use of I/O buffers in order to minimize the number of disk accesses. Also, we have designed a mechanism to overlap I/O operations with computation that allows reading and writing in parallel when two different hard disks are employed. As a result of these optimizations, the program time (i.e. wall time) approaches the reconstruction time. With regard to the second topic, we have elaborated a scheme which dynamically assigns more workload to the fastest threads, hence adapting itself to the system where it runs. This fact leaves the door open to use our scheme in heterogeneous architectures, i.e. those equipped with devices with different computing power. In addition, the tomographic reconstruction problem is also susceptible to application of more sophisticated dynamic load balancing techniques developed in the HPC field.

The approach to tomographic reconstruction discussed here does not need any special hardware (e.g. GPU or cluster) to run: just a standard computer with a multicore processor is required. This facilitates the distribution and usage of the software (i.e. the user will not have to worry about libraries and will not have to deal with a cluster). Furthermore, the comparative study carried out here has revealed that our approach outperforms a standard package in the field. It is thus expected to be very useful in laboratories of structural biology as the people who work there demand fast and easy to manage software solutions.

In the future, this work could be extended by using the new SIMD instructions introduced by Intel in their most modern processors. These instructions are known as AVX (Advanced Vector eXtensions) and are able to process eight single-precision floating-point numbers at a time. This would let us reconstruct eight slices at once, therefore potentially doubling the speed of our algorithms. On the other hand, many of the optimizations analysed here could be applied to other reconstruction algorithms (e.g. ART (Algebraic Reconstruction Technique)), other operations involved in the ET image processing workflow (e.g. noise reduction) or other scientific problems that also require a significant processing time.

## Supporting Information

Figure S1 **Description of the optimization procedure on multicore computers**. (left) Flowchart of the optimization procedure. There are two blocks of optimizations. In the first one, the basic optimizations intend to speed up the code on a single CPU core. Then, code modifications to exploit vector processing are made. Going back and forth between the basic optimizations and vectorization is often needed for fine code tuning (e.g. to optimize access to cache memory to read/write data vectors). The second block of optimizations intends to take advantage of the power of the multiple CPU cores available in the computer. The first set of modifications here relies on multithreading, which splits the general problem into tasks that are then mapped and executed in parallel on the different cores. The second set then focuses on disk access optimization, though this step is closely related to the previous one. (right) sketch of a computer architecture based on multicore processors. (bottom-right) Modern computers ship with several multicore chips (typically 2–4) configured to share a centralized memory. Each multicore chip contains several computing CPU cores (2–8) sharing a cache memory (typically the third level, L3). (top-right) Internally, each single CPU core consists of several functional units (FUs) that execute the scheduled micro-instructions. The basic optimizations intend to maximize the use of FUs, minimize the latencies and waiting gaps of micro-instructions, and guarantee an optimum exploitation of cache memory (typically, two levels within the CPU core). One of the FUs is the vector unit, which follows the SIMD execution model shown in Figure 5 of the main text. Vectorization aims to make the most of the vector unit by performing the same operation on data vectors.
(TIF)

Table S1 **I/O analysis.**
(PDF)

## References

[1] Herman GT (2009) Fundamentals of Computerized Tomography: Image Reconstruction from Projections. London: Springer, second edition.

[2] Fernández JJ (2012) Computational methods for electron tomography. Micron 43: 1010–1030. 22658288 10.1016/j.micron.2012.05.003

[3] Fernández JJ, Sorzano COS, Marabini R, Carazo JM (2006) Image processing and 3-D reconstruction in electron microscopy. IEEE Signal Processing Magazine 23: 84–94.

[4] Frank J, editor (2006) Electron Tomography: Methods for Three-Dimensional Visualization of Structures in the Cell. New York: Springer, second edition.

[5] Lučić V, Förster F, Baumeister W (2005) Structural studies by electron tomography: from cells to molecules. Annual Review of Biochemistry 74: 833–865. 10.1146/annurev.biochem.73.011303.074112 15952904

[6] Beck M, Lučić V, Förster F, Baumeister W, Medalia O (2007) Snapshots of nuclear pore complexes in action captured by cryo-electron tomography. Nature 449: 611–615. 17851530 10.1038/nature06170

[7] Brandt F, Etchells SA, Ortiz JO, Elcock AH, Hartl FU, et al (2009) The native 3D organization of bacterial polysomes. Cell 136: 261–271. 19167328 10.1016/j.cell.2008.11.016

[8] Grünewald K, Desai P, Winkler DC, Heymann JB, Belnap DM, et al (2003) Three-dimensional structure of herpes simplex virus from cryo-electron tomography. Science 302: 1396–1398. 14631040 10.1126/science.1090284

[9] He W, Ladinsky MS, Huey-Tubman KE, Jensen GJ, McIntosh JR, et al (2008) FcRn-mediated antibody transport across epithelial cells revealed by electron tomography. Nature 455: 542–546. 18818657 10.1038/nature07255 PMC2773227

[10] Li S, Fernández JJ, Marshall WF, Agard DA (2012) Three-dimensional structure of basal body triplet revealed by electron cryo-tomography. EMBO J 31: 552–562. 22157822 10.1038/emboj.2011.460 PMC3273388

[11] Medalia O, Weber I, Frangakis AS, Nicastro D, Gerisch G, et al (2002) Macromolecular architecture in eukaryotic cells visualized by cryoelectron tomography. Science 298: 1209–1213. 12424373 10.1126/science.1076184

[12] Fernández JJ (2008) High performance computing in structural determination by electron cryomicroscopy. J Struct Biol 164: 1–6. 18675361 10.1016/j.jsb.2008.07.005

[13] Perkins GA, Renken CW, Song JY, Frey TG, Young SJ, et al (1997) Electron tomography of large, multicomponent biological structures. J Struct Biol 120: 219–227. 9441927 10.1006/jsbi.1997.3920

[14] Fernández JJ, García I, Carazo JM, Marabini R (2007) Electron tomography of complex biological specimens on the Grid. Future Generation Computer Systems 23: 435–446.

[15] Lee D, Lin AW, Hutton T, Akiyama T, Shinji S, et al (2003) Global telescience featuring IPv6 at iGrid2002. Future Generation Computer Systems 19: 1031–1039.

[16] Peltier ST, Lin AW, Lee D, Mock S, Lamont S, et al (2003) The Telescience Portal for advanced tomography applications. J Parallel Distrib Comput 63: 539–550.

[17] Fernández JJ, Carazo JM, García I (2004) Three-dimensional reconstruction of cellular structures by electron microscope tomography and parallel computing. J Parallel Distrib Comput 64: 285–300.

[18] Fernández JJ, Gordon D, Gordon R (2008) Efficient parallel implementation of iterative reconstruction algorithms for electron tomography. J Parallel Distrib Comput 68: 626–640.

[19] Fernández JJ, Lawrence AF, Roca J, García I, Ellisman MH, et al (2002) High-performance electron tomography of complex biological specimens. J Struct Biol 138: 6–20. 12160697 10.1016/s1047-8477(02)00017-5

[20] Fritzsche PC, Fernández JJ, Rexachs D, García I, Luque E (2010) Analytical performance prediction for iterative reconstruction techniques in electron tomography of biological structures. International Journal of High Performance Computing Applications 24: 457–468.

[21] Wan X, Zhang F, Liu Z (2009) Modified simultaneous algebraic reconstruction technique and its parallelization in cryo-electron tomography. In: Proceedings of the 15th International Conference on Parallel and Distributed Systems (ICPADS 2009): 384–390.

[22] Zheng SQ, Keszthelyi B, Branlund E, Lyle JM, Braunfeld MB, et al (2007) UCSF tomography: An integrated software suite for real-time electron microscopic tomographic data collection, alignment, and reconstruction. J Struct Biol 157: 138–147. 16904341 10.1016/j.jsb.2006.06.005

[23] Kirk D, Hwu WW (2010) Programming Massively Parallel Processors: A Hands-on Approach. Morgan Kaufmann.

[24] Castaño-Díez D, Moser D, Schoenegger A, Pruggnaller S, Frangakis AS (2008) Performance evaluation of image processing algorithms on the GPU. J Struct Biol 164: 153–160. 18692140 10.1016/j.jsb.2008.07.006

[25] Castaño-Díez D, Mueller H, Frangakis AS (2007) Implementation and performance evaluation of reconstruction algorithms on graphics processors. J Struct Biol 157: 288–295. 17029985 10.1016/j.jsb.2006.08.010

[26] Palenstijn WJ, Batenburg KJ, Sijbers J (2011) Performance improvements for iterative electron tomography reconstruction using graphics processing units (GPUs). J Struct Biol 176: 250–253. 21840398 10.1016/j.jsb.2011.07.017

[27] Vázquez F, Garzón EM, Fernández JJ (2010) A matrix approach to tomographic reconstruction and its implementation on GPUs. J Struct Biol 170: 146–151. 20132889 10.1016/j.jsb.2010.01.021

[28] Vázquez F, Garzón EM, Fernández JJ (2011) Matrix implementation of simultaneous iterative reconstruction technique (SIRT) on GPUs. The Computer Journal 54: 1861–1868.

[29] Xu W, Xu F, Jones M, Keszthelyi B, Sedat J, et al (2010) High-performance iterative electron tomography reconstruction with long-object compensation using graphics processing units (GPUs). J Struct Biol 171: 142–153. 20371381 10.1016/j.jsb.2010.03.018 PMC2885506

[30] Wan X, Zhang F, Chu Q, Liu Z (2011) High-performance blob-based iterative reconstruction of electron tomography on multi-GPUs. Lect Notes in Comput Sci 6674: 61–72.

[31] Wan X, Zhang F, Chu Q, Liu Z (2012) High-performance blob-based iterative three-dimensional reconstruction in electron tomography using multi-GPUs. BMC Bioinformatics (Suppl 10): S4. 10.1186/1471-2105-13-S10-S4 PMC3382438 22759428

[32] Zheng SQ, Branlund E, Kesthelyi B, Braunfeld MB, Cheng Y, et al (2011) A distributed multi-GPU system for high speed electron microscopic tomographic reconstruction. Ultramicroscopy 111: 1137–1143. 21741915 10.1016/j.ultramic.2011.03.015 PMC3190582

[33] Agulleiro JI, Vazquez F, Garzon EM, Fernandez JJ (2012) Hybrid computing: CPU+GPU coprocessing and its application to tomographic reconstruction. Ultramicroscopy 115: 109–114. 22475372 10.1016/j.ultramic.2012.02.003

[34] Hennessy JL, Patterson DA (2011) Computer architecture: a quantitative approach. Morgan Kaufmann, fifth edition.

[35] Lee VW, Kim C, Chhugani J, Deisher M, Kim D, et al (2010) Debunking the 100X GPU vs. CPU myth: an evaluation of throughput computing on CPU and GPU. ACM SIGARCH Computer Architecture News 38: 451–460.

[36] Agulleiro JI, Garzón EM, García I, Fernández JJ (2010) Vectorization with SIMD extensions speeds up reconstruction in electron tomography. J Struct Biol 170: 570–575. 20085820 10.1016/j.jsb.2010.01.008

[37] Agulleiro JI, Fernández JJ (2011) Fast tomographic reconstruction on multicore computers. Bioinformatics 27: 582–583. 21172911 10.1093/bioinformatics/btq692

[38] Beeby M, Cho M, Stubbe J, Jensen GJ (2012) Growth and localization of polyhydroxybutyrate granules in Ralstonia eutropha. J Bacteriol 194: 1092–1099. 22178974 10.1128/JB.06125-11 PMC3294789

[39] Briegel A, Li X, Bilwes AM, Hughes KT, Jensen GJ, et al (2012) Bacterial chemoreceptor arrays are hexagonally packed trimers of receptor dimers networked by rings of kinase and coupling proteins. Proc Natl Acad Sci USA 109: 3766–3771. 22355139 10.1073/pnas.1115719109 PMC3309718

[40] Badia-Martinez D, Peralta B, Andres G, Guerra M, Gil-Carton D, et al (2012) Three-dimensional visualization of forming hepatitis c virus-like particles by electron-tomography. Virology 430: 120–126. 22657942 10.1016/j.virol.2012.05.001

[41] Ounjai P, Kim KD, Lishko PV, Downing KH (2012) Three-dimensional structure of the bovine sperm connecting piece revealed by electron cryotomography. Biol Reprod 87: 73, 1–9. 10.1095/biolreprod.112.101980 PMC3464910 22767409

[42] Murat D, Falahati V, Bertinetti L, Csencsits R, Kornig A, et al (2012) The magnetosome membrane protein, MmsF, is a major regulator of magnetite biomineralization in Magnetospirillum magneticum AMB-1. Mol Microbiol 85: 684–699. 22716969 10.1111/j.1365-2958.2012.08132.x PMC3570065

[43] Perez-Berna AJ, Ortega-Esteban A, Menendez-Conejero R, Winkler DC, Menendez M, et al (2012) The role of capsid maturation on adenovirus priming for sequential uncoating. J Biol Chem 287: 31582–31595. 22791715 10.1074/jbc.M112.389957 PMC3438990

[44] Radermacher M (2006) Weighted back-projection methods. In: Frank J, editor, Electron Tomography: Methods for Three-Dimensional Visualization of Structures in the Cell, Springer. Second edition, 245–273.

[45] Gilbert P (1972) Iterative methods for the three-dimensional reconstruction of an object from projections. J Theor Biol 36: 105–117. 5070894 10.1016/0022-5193(72)90180-4

[46] Cyrklaff M, Risco C, Fernández JJ, Jiménez MV, Esteban M, et al (2005) Cryo-electron tomography of vaccinia virus. Proc Natl Acad Sci USA 102: 2772–2777. 15699328 10.1073/pnas.0409825102 PMC549483

[47] Wadleigh KR, Crawford IL (2000) Software Optimization for High Performance Computing. Prentice Hall PTR.

[48] Frigo M, Johnson SG (2005) The design and implementation of FFTW3. Proceedings of the IEEE 93: 216–231.

[49] Kremer J, Mastronarde D, McIntosh JR (1996) Computer visualization of three-dimensional image data using imod. J Struct Biol 116: 71–76. 8742726 10.1006/jsbi.1996.0013

