# Supplementary figures and images for: Evaluation of a Multicore-Optimized Implementation for Tomographic Reconstruction
Source: PLoS One. 2012 Nov 6;7(11):e48261. doi: 10.1371/journal.pone.0048261 (PMC3491071; doi:10.1371/journal.pone.0048261)

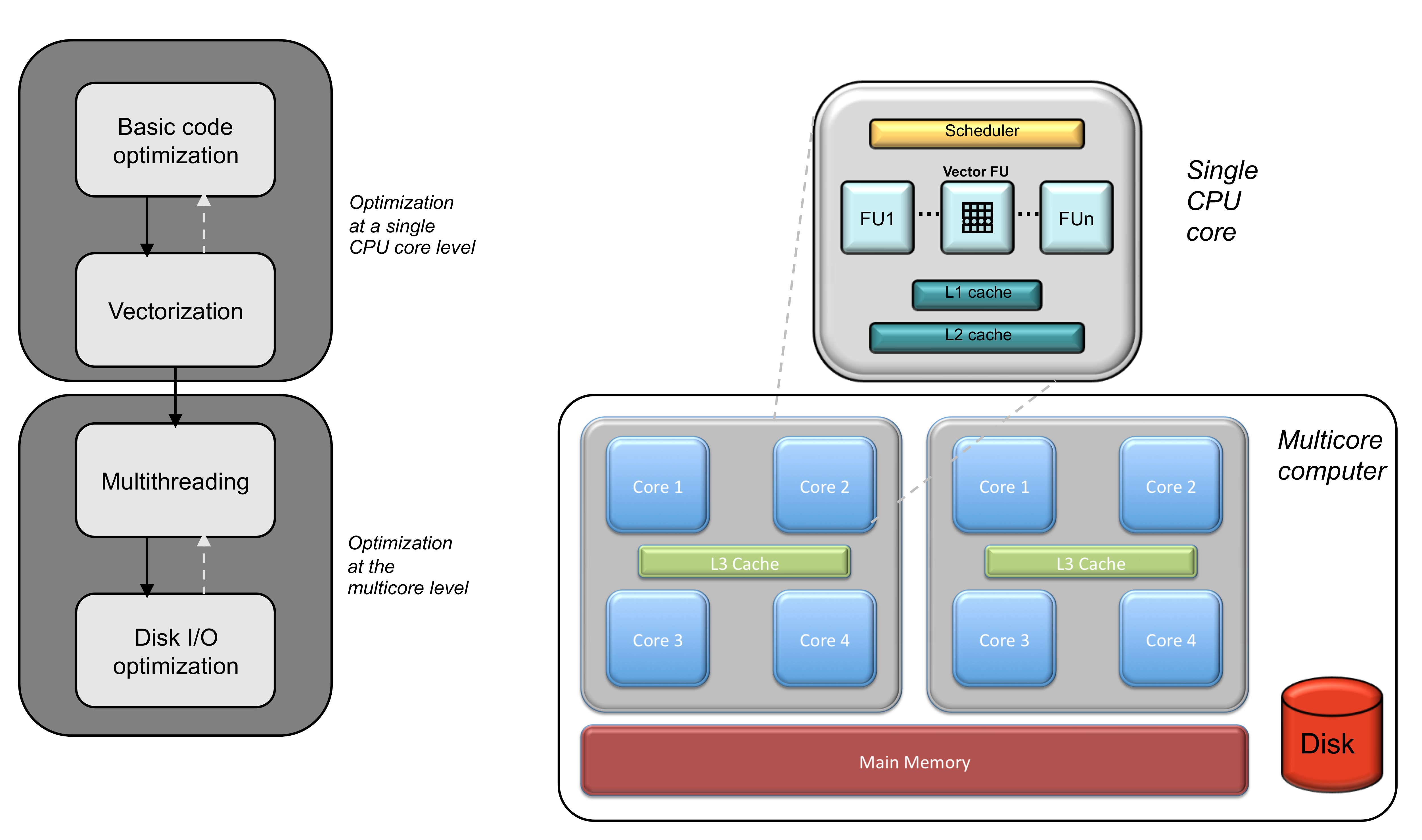

Supplement: Figure S1 — Description of the optimization procedure on multicore computers. (left) Flowchart of the optimization procedure. There are two blocks of optimizations. In the first one, the basic optimizations intend to speed up the code on a single CPU core. Then, code modifications to exploit vector processing are made. Going back and forth between the basic optimizations and vectorization is often needed for fine code tuning (e.g. to optimize access to cache memory to read/write data vectors). The second block of optimizations intends to take advantage of the power of the multiple CPU cores available in the computer. The first set of modifications here relies on multithreading, which splits the general problem into tasks that are then mapped and executed in parallel on the different cores. The second set then focuses on disk access optimization, though this step is closely related to the previous one. (right) sketch of a computer architecture based on multicore processors. (bottom-right) Modern computers ship with several multicore chips (typically 2–4) configured to share a centralized memory. Each multicore chip contains several computing CPU cores (2–8) sharing a cache memory (typically the third level, L3). (top-right) Internally, each single CPU core consists of several functional units (FUs) that execute the scheduled micro-instructions. The basic optimizations intend to maximize the use of FUs, minimize the latencies and waiting gaps of micro-instructions, and guarantee an optimum exploitation of cache memory (typically, two levels within the CPU core). One of the FUs is the vector unit, which follows the SIMD execution model shown in Figure 5 of the main text. Vectorization aims to make the most of the vector unit by performing the same operation on data vectors. (TIF) [file pone.0048261.s001.tif]
