# Supplementary material for: Evaluation of a Multicore-Optimized Implementation for Tomographic Reconstruction
Source: PLoS One. 2012 Nov 6;7(11):e48261. doi: 10.1371/journal.pone.0048261 (PMC3491071; doi:10.1371/journal.pone.0048261)
Supplement: Table S1 — I/O analysis. (PDF) [file pone.0048261.s002.pdf]

Table S1. I/O analysis

WBP					
**Static scheme**					
Buffer	*16*	*32*	*64*	*128*	*256*
$T_{rec.}$	44.00	43.52	43.18	43.87	46.22
$T_{I/O}$	167.95	130.56	105.04	102.17	110.93
$T_{prog.}$	212.73	174.70	147.80	146.40	157.29
Mem.	0.40	0.72	1.40	2.60	5.20
**Dynamic scheme**					
Buffer	*16*	*32*	*64*	*128*	*256*
$T_{rec.}$	49.66	48.25	47.51	47.50	46.97
$T_{I/O}$	165.32	110.13	85.48	79.56	62.57
$T_{prog.}$	218.10	161.75	137.76	129.37	111.24
Mem.	0.16	0.24	0.40	0.72	1.40
**Asynchronous I/O (1 disk)**					
Buffer	*16*	*32*	*64*	*128*	*256*
$T_{rec.}$	51.54	54.17	54.36	54.19	53.52
$T_{I/O}$	192.81	133.82	78.43	69.72	63.31
$T_{prog.}$	244.97	188.68	133.47	124.57	117.48
Mem.	0.16	0.24	0.40	0.72	1.40
**Asynchronous I/O (2 disks)**					
Buffer	*16*	*32*	*64*	*128*	*256*
$T_{rec.}$	49.88	53.13	52.98	53.41	54.01
$T_{I/O}$	103.42	70.49	38.17	14.61	12.45
$T_{prog.}$	153.92	124.28	91.83	68.72	67.12
Mem.	0.16	0.24	0.40	0.72	1.40

SIRT					
**Static scheme**					
Buffer	*16*	*32*	*64*	*128*	*256*
$T_{rec.}$	461.24	461.52	461.99	470.99	464.10
$T_{I/O}$	23.86	22.66	23.91	28.49	68.89
$T_{prog.}$	489.38	488.29	488.71	502.94	535.26
Mem.	0.42	0.74	1.40	2.60	5.20
**Dynamic scheme**					
Buffer	*16*	*32*	*64*	*128*	*256*
$T_{rec.}$	466.03	465.91	464.60	463.79	465.74
$T_{I/O}$	51.89	40.15	35.80	43.26	45.23
$T_{prog.}$	535.05	521.90	515.62	521.06	520.88
Mem.	0.18	0.26	0.42	0.74	1.40
**Asynchronous I/O (1 disk)**					
Buffer	*16*	*32*	*64*	*128*	*256*
$T_{rec.}$	460.79	470.99	470.67	471.00	470.78
$T_{I/O}$	130.86	7.79	3.07	3.20	4.86
$T_{prog.}$	593.57	480.45	475.41	475.82	477.28
Mem.	0.18	0.26	0.42	0.74	1.40
**Asynchronous I/O (2 disks)**					
Buffer	*16*	*32*	*64*	*128*	*256*
$T_{rec.}$	463.20	470.47	470.16	469.93	471.26
$T_{I/O}$	106.66	3.67	3.20	3.06	4.27
$T_{prog.}$	571.85	475.79	475.00	474.58	477.13
Mem.	0.18	0.26	0.42	0.74	1.40

I/O analysis for the volume 2048×512×2048. $T_{rec.}$, $T_{I/O}$ and $T_{prog.}$ denote the reconstruction time, the disk I/O time and the actual program time, respectively. Note that $T_{rec.}$ is slightly higher in the asynchronous I/O since the cores are shared between workers and I/O threads. Nonetheless, the I/O time reduction outweighs this little increment.
